# Essential components of an educational program for implementing skin-to-skin contact for preterm infants in intensive care units: an integrative literature review

**DOI:** 10.1186/s12884-024-06447-6

**Published:** 2024-04-16

**Authors:** Takalani T. Denge, Nokwanda Edith Bam, Welma Lubbe, Annah Rakhudu

**Affiliations:** 1 NuMIQ (Quality in Nursing and Midwifery), Faculty of Health Sciences, North-West University, Potchefstroom, South Africa; 2 NuMIQ (Quality in Nursing and Midwifery), Faculty of Health Sciences, North-West University, Mafikeng, South Africa

**Keywords:** Educational program, Preterm infants, Parents, Intensive care units, Skin-to-skin contact

## Abstract

**Background:**

Globally, prematurity is the primary factor behind the mortality of children under the age of 5 years, resulting in approximately 1 million children dying annually. The World Health Organization (WHO) recommends Skin-to-Skin Contact (SSC) as part of routine care for preterm infants. Evidence shows that SSC reduces mortality, possibly by improving thermoregulation, facilitating the earlier initiation of breastfeeding and reducing the risk of nosocomial infection. An educational program for implementing SSC has been demonstrated to enhance the knowledge and practice of parents and nurses in intensive care units. This study, the first of its kind in the North West Province (NWP), aims to identify the essential components of an educational program for implementing SSC for premature infants in intensive care units.

**Objective:**

This paper presents an integrative literature review that critically synthesizes research-based literature on essential components of an educational program for implementing SSC for preterm infants in intensive care units.

**Methods:**

A comprehensive search of electronic databases, such as CINAHL, MEDLINE, PsycINFO, ProQuest and Health Source: Nursing/Academic Edition and Health Source-Consumer Edition, was conducted using different keywords and references lists from the bibliography.

**Results:**

Twelve articles relevant to this review were identified, read and synthesized to answer the research question. Three essential components emerged from the findings of this review, namely (1) the necessity of policy and role players for implementing SSC, (2) the availability of education and training, and (3) counseling and support for parents of preterm infants.

**Conclusions:**

The outcomes of this study have the potential to facilitate the implementation and expansion of SSC in intensive care units. This could aid program implementers, policymakers, and researchers to implement and scale up this important tool in intensive care units.

## Introduction and background

An estimated 13, 4 million infants were delivered prematurely in 2020, indicating that more than 1 to 10 births are premature [1]. Up to 70% of neonatal deaths occur in preterm or Low Birth Weight (LBW) infants within the first 3 days after birth, but the mortality can be reduced by effective newborn care [1]. Although progress has been made in reducing infant mortality rate globally, more than 45% of the five million under-five mortality rate caters for infants death in the first months of life and approximately one million of these children die within the first 28 days from prematurity and LBW [2]. According to the World Health Organization (WHO), a preterm infant is infant born after the first day of the last menstrual period but before the completion of 37 weeks of gestation; that is, they have a small gestational age and LBW [3].

In South Africa, the neonatal mortality rate in 2017 was reported as 12 per 1000 live births [4]. Moreover, approximately 10% of the preterm infants born in South Africa do not survive and this could be viewed as a serious threat to childhood outcomes [4]. Addressing the global burden of preterm birth is essential for reducing preterm-related neonatal and child mortality and achieving the Sustainable Development Goal (SDG) target 3.2 (which commits to reducing neonatal mortality to 12 or fewer deaths per 1000 livebirths [5]. Therefore, the WHO published new recommendations on the care of preterm infants, such as skin-to-skin contact (SSC), in order to reduce mortality in preterm and low-birth-weight babies [3].

SSC has been shown to have positive effects on preterm infants in intensive care units. Several studies found that it is associated with a reduction in neonatal mortality rate, improved growth, neurodevelopmental outcomes, and breastfeeding [6, 7]. SSC has been shown to alleviate maternal anxiety, reshape the maternal role, promote active breast pumping, and build maternal confidence in the care of the baby. Therefore, SSC should be considered as a standard of care for preterm infants, as it provides numerous benefits for both infants and mothers.

Implementing an educational program for SSC has been shown to improve the knowledge and practices of parents and nurses with preterm infants in intensive care units [8, 9]. In addition, Ragad et al. further stated that virtual education and supportive programs improve mothers’ resilience with preterm infants in intensive care units [10]. Nurses’ knowledge and provision of SSC can be improved through structured training programs, which address barriers to early initiation of SSC in preterm infants [11]. Therefore, continuous educational programs are recommended to increase awareness and ensure sufficient knowledge and practice of SSC among parents of preterm infants.

An educational program to implement SSC and early breastfeeding in a rural hospital in Mexico was developed using a two-step approach. This study found that a simple and low-cost educational program resulted in SSC and early breastfeeding being included as part of standard care in a rural hospital [12]. This type of program is essential because mothers of preterm infants need specific information, since the type of information aimed parents in general does not meet their specific needs.

Researchers of a study conducted in Canada found that a digital educational program improved parents’ knowledge and fostered exchanges between parents and nurses [13]. A similar result was achieved in a study conducted by Mukarubayiza and Gowan [14] at a Kigali district hospital in Rwanda; they concluded that an educational program was effective in improving parents’ knowledge of caring for preterm infants. In addition, Astute and Pandin [15] highlighted that choosing the right educational method can improve the ablitiy of parents to properly care for and provide developmental care for preterm infants.

Although very limited published literature exists about educational programs regarding the care of preterm infants in intensive care units in Low to Middle-Income Countries (LMICs), there is global consensus that such countries have an urgent need to implement equitable and sustainable programs for vulnerable families. [16, 17]. Since the premature birth population is often associated with vulnerable families, there is an urgent need to implement equitable and sustainable program for the care of preterm infants. Therefore, the no studies regarding the essential components of educational programs to implement SSC for preterm infants in intensive care units could be identified in SA, especially in the North West Province (NWP). Thus, this paper aims to conduct an integrative literature review that synthesizes research-based literature on the essential components of an educational program to implement SSC for preterm infants in intensive care units.

## Materials and methods

The researcher adopted an Integrative Literature Review (ILR) approach to answer a review question. The ILR is a method of research that explores, critically appraises, synthesizes and presents the findings on available literature regarding a review question. In this instance that question considers the essential components of an educational program to implement SSC for preterm infants in intensive care units [18]. ILR allows for simultaneous inclusion of quantitative and qualitative data for both experimental and non-experimental studies and mixed method studies [19]. In addition, the approach incorporates a wide range of purposes: to define concepts, to review theories, to review evidence, and to analyse methodological issues of a particular topic [19].

## Steps of integrative literature review

**Fig. 1:**
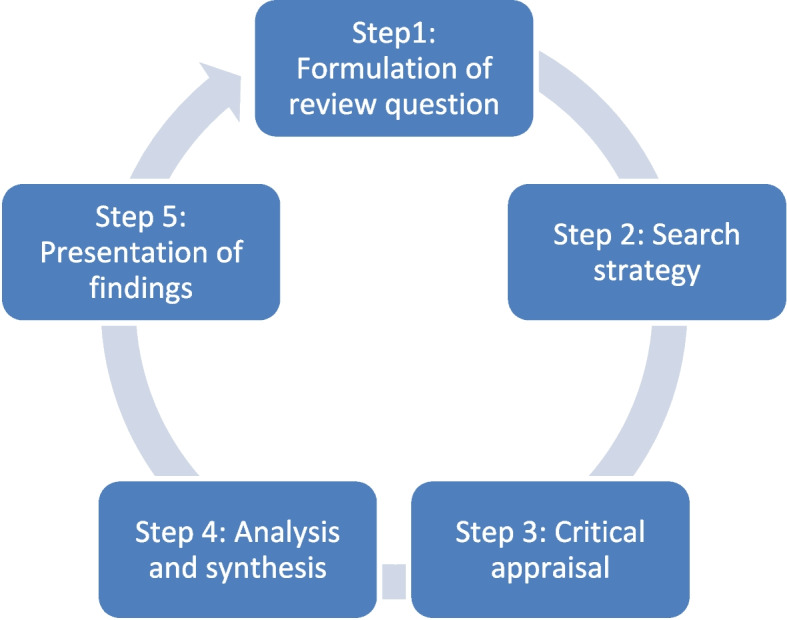
The five steps of ILR adopted in this study [16]

The ILR employed the framework [20], by expanding on the data analysis and synthesis stage to enhance the systematic nature and rigour of the process. The following five steps were used: review question, search strategy, critical appraisal, data analysis and synthesis (result discussion of the critical appraisal) and conclusion statement. These steps were discussed in detail with the results obtained from each step. Figure 1 provides a visual presentation of the steps.

### Step 1: Formulation of the review question

The review question refers to the problem formulation, starting with a clear identification of the problem statement, definitions, and formulation of the review question to address the review purpose [20]. The review question was constructed by utilizing the population, intervention, outcome, and timeframe (PIOT) format as indicated in Table 1 [20]. For the purpose of this study, the population (P) refers to parents of preterm infants. The interventions (I) comprised essential components of an educational program needed for parents with preterm infants admitted in the intensive care units to implement SSC. Indicators for health and wellbeing of both parents and preterm infants were set as the outcome (O) measure. The time frame (T) was time spent by preterm infants in the intensive care units. Based on the outcomes of the PIOT, the following review question was formulated: “What is stated in published literature regarding the key components of an educational program to implement SSC for preterm infants admitted in the intensive care units?” Below is a table indicating the PIOT format [20] (Table 1).

**Table 1:** The PIOT format adopted in this study [20]

P	I	O	T
Parents/mothers of preterm infants	Essential components of educational program to implement SSC	Health and wellbeing of both parents and preterm infants	Time that preterm infants spent in the intensive care units

### Step 2: Search strategy

The second step in the review process was to develop a search strategy. The inclusion criteria for this search were all national and international literature (research and non-research) on essential components of an educational program to implement SSC for preterm infants admitted in intensive care units, published from 2019 to 2023. Only studies written in English were included, since it is the language shared by all the review team members and resources for translation services were limited. The studies included in this review used qualitative, quantitative and mixed methods, and thus included a systematic review of documents, interventions, reports, components or strategies, reviews, theses, and dissertations.

#### Exclusion criteria

Prefaces, letters to editors and editorials were excluded from the search since they represent opinions and are not regarded as primary research. Duplicated articles were also excluded after deciding which one (if they were not identical) provided the most comprehensive data regarding the study. During the search stage of the ILR, studies or documents that did not discuss essential components of an educational program in the title, abstract or text were excluded. The title of each study or article was read to determine its relevance. Studies or articles were excluded if they were not accessible to the researcher via the university’s library and inter-lending options.

#### Literature search

Studies were collected using multiple data platforms, while a clear description of inclusion and exclusion criteria guided the search process. The search was conducted from February to June 2023 on the following electronic reference databases: CINAHL, MEDLINE, PsycINFO, ProQuest, Health Source – Nursing/Academic Edition, and Health Source – Consumer Edition. Different keywords were used as well as the reference lists from the bibliography of the documents sampled during the previous stages of the study.

**Fig. 2:**
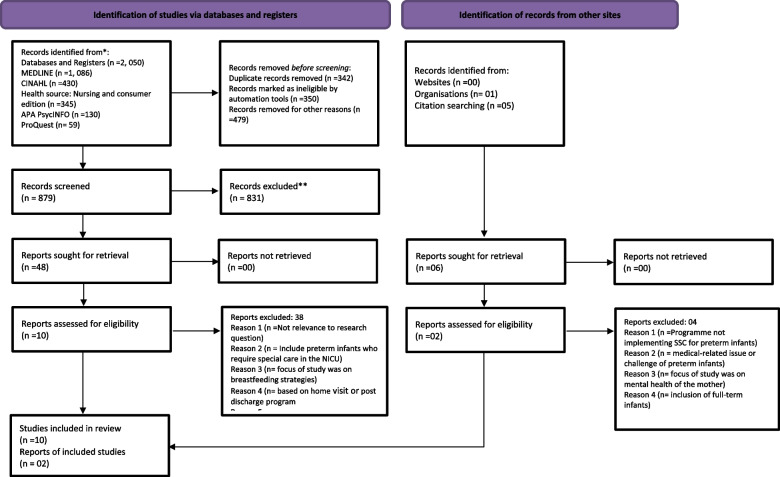
PRISMA diagram for retrieval strategies and exclusion criteria [21]

The search strategy followed the phrase approach of combining any search terms and keywords with “AND”, “OR”, and the wild card symbol (*) to yield more relevant results. The following search phrases and keywords were used: essential components of an educational program OR intervention AND skin-to-skin contact OR care OR kangaroo-mother care AND parents* OR mothers AND preterm infants* OR premature AND infant or baby* AND intensive care units OR neonate units. A total of 2,050 records were identified. Along with the electronic database search, a manual search of selected records was conducted to search for additional documents or studies that could be considered for inclusion. Figure 2 presents a PRISMA (Preferred Reporting Items for Systematic reviews and Meta-Analyses) flow diagram to illustrate the process of identifying and selecting studies for inclusion in the review [21].

### Step 3: Critical appraisal

The third step in the ILR process entailed an in-depth appraisal of the relevant documents that were identified during the searches. A critical appraisal was needed to identify any low-quality documents that could be excluded based on the basis of methodological quality and rigour; this would strengthen the evidence included in the final ILR [22]. In the critical appraisal process, the authors evaluated all the components of each study, including the introduction, methods, findings, and discussion. The trustworthiness, credibility, congruence, and transferability of the qualitative studies’ findings were carefully and systematically determined [23]. Content validity in quantitative studies was assessed to ensure the developed self-reported instruments to collect data adequately represented the intended scope of the study [23].

The methodological quality of all 12 selected studies was assessed by means of a checklist in the form of the critical appraisal tool: Standard Quality Assessment Criteria for Evaluating Primary Research Papers from a Variety of Fields, which was developed by Kmet et al. [22]. The 12 studies selected for critical appraisal were assessed by authors, who have experience in the ILR of studies for methodological rigor. The procedure required the authors to check responses (yes, no, N/A) against the 10-question checklist to determine whether a study would be included or not. Scores were interpreted based on the recommended criteria: 50–100 percent denoted a “good” study, while less than 50 percent indicated a “poor” study. Studies with a score below 50 percent were excluded. After discussion, the authors were able to agree on the selected studies to be included. All articles were ranked based on the review guidelines of the tool. The results of the critical appraisal revealed that 12 studies were suitable for inclusion in the review. Table 2 presents the quality score of each of the studies included.

**Table 2:** Quality score of articles included

Author	Year	Country	Quality score (100%)
Medhanyie et al	2019	Ethopia and India	**97,6**
Nuraini et al	2019	Indonesia	**80%**
Sanchez-Espino et al	2019	Mexico	**85,2%**
Lui et al	2020	China	**96,4%**
Maniago et al	2020	14 diverse Countries (Australia, Brazil, Denmark, Norway, Sweden, Canada, USA, Estonia, Iran, Finland, Norway, Spain and Sweden)	**99,6%**
WHO	2020	South Asia and sub-Sub-Saharan Africa (Ghana, India, Malawi, Nigeria, and Tanzania)	**92,8%**
Almutairi	2022	Saudi Arabia	**85,7%**
Fatma et al	2022	Egypt	**90,9%**
Muttau et al	2022	Zambia	**92,5%**
Habte et al	2023	Southern Ethopia	**100%**
Moran et al	2023	High-income settings	**89%**

### Step 4: Data analysis and synthesis

Throughout the critical appraisal phase that revealed 12 studies suitable for inclusion in the review, the literature was read and re-read several times. In stage four of the review, characteristics of 12 selected studies, including the citation details (authors, year, country of publication), aims, sample, results, and findings relevant to the review question were captured and are illustrated in Table 3

**Table 3:** Characteristics of selected studies

Author, year	Study design	Context	Aim of the study	Sample		Results/Findings	Results/Findings relevant to review question
Medhanyie et al. 2019 [24]	Mixed-methods design	Ethopia and India	The aim was to develop and evaluate district-level models for scaling up Kangaroo Mother Care (KMC) in India and Ethopia that could achieve high-population coverage	Sampling of babies with birth weight under 2000 g		The findings identified barriers and contextual factors that affect implementation and utilization of KMC and design to scalable models to deliver KMC across the facility – community continuum. Implementation and evaluation of these models occurred at three levels: pre-KMC facility – to maximize the number of newborns getting to a facility that provides KMC; KMC facility-for initiation and maintenance of KMC, and post – KMC facility-for continuation of KMC at home	Barriers and enablers of SSC in mothers and nurses; Counselling mothers on SSC and breastfeeding
Nuraini et al. 2019 [25]	Qualitative approach	Indonesia	The aim was to assess the skills of neonatal nurses one year after KMC training in the Pasar Rebo District General Hospital	Purposive sampling		A year after follow-up, there was neither KMC training nor a clear policy. It was mentioned that there was a standard operating procedure (SOP) of KMC and routine transfer of health personnel. However, there were no free KMC gowns to support the training	KMC training Policy and procedure regarding KMC
Sanchez-Espino et al. 2019 [12]	A two-step educational intervention	Mexico	The aim was to assess if a dual educational intervention in a rural hospital in Mexico could modify current practice and accomplish early SSC and early breastfeeding	Labour and birthing staff was sample in the first step. Second step was all pregnant women with uncomplicated pregnancies at 36 weeks’ of gestation		A total of 142 births met inclusion criteria. Of those, 109 received SSC and early breastfeeding. The average time of initiation of SSC in the first and last month of the study was 18.5 and 9.6 min of life. The average duration of SSC in the first and last month was 22 and 40.9 min. The average time of onset of breastfeeding in the first and last month was 48.9 and 34.4 min of life	Educate mothers, registered nurses and midwives on the benefits of early SSC and breastfeeding
Lui et al. 2020	A cross-sectional study	China	The aim was to investigate the feasibility and parental experience of adopting KMC in a Chinese context by studying the implementation of a KMC program in neonatal intensive care units (NICUs)	Eight NICUs were purposively selected		135 preterm infants received KMC, 21,1% of all preterm infants were discharged. 94,8% of parents who participated in the survey indicated that KMC was positively accepted by their family members; 60,4% of the parents claimed that KMC could relieve anxiety, and 57,3% claimed it prompted more interactions with medical staff, while 69.8% suggested it increased parental confidence in their ability to care for their infants	Training and support implementation of SSC: Information regarding the concept of SSC was given to promote parental knowledge
Maniago et al. 2020 [26]	Integrative literature review	14 diverse Countries (Australia, Brazil, Denmark, Norway, Sweden, Canada, USA, Estonia, Iran, Finland, Norway, Spain, and Sweden)	The aim of the ILR was to critically analyze data extracted from existing primary research and explore nurses’ barriers in implementing KMC in order to illustrate directions for future research. It also explored strategies to reduce barriers to KMC implementation	Purposive sampling of nurses		The search revealed 19 articles from diverse countries. Four main themes generated from the synthesis of the findings: (i) barriers related to nurses perspectives and emotion towards KMC, (ii) healthcare institution barriers towards KMC (iii) barriers related to parental experience in providing KMC and (iv) strategy to improve KMC implementation	Education of mothers on benefits of SSC and implementation of guidelines to promote SSC Newborn and mothers’ needs Training program to reduce perceived barrier and increase SSC practice
WHO 2020 [3, 27]	Randomized controlled trial	South Asia and sub-Sub-Saharan Africa (Ghana, India, Malawi, Nigeria, and Tanzania)	The aim was to evaluate the safety and efficacy of continuous KMC initiated as soon as possible after birth compared with the current recommendation of initiating continuous KMC after stabilization in neonates with a birth weight between 1,0 and less then 1,8 kg		Eligible participants were randomly assigned to intervention and control group	The intervention resulted in an important enhancement of the LMIC settings in which mothers are not separated from their baby in neonatal intensive care units	Promotion and support for continuous SSC; provision of health care for both mother and preterm infants; promotion and support of breastfeeding
Almutairi. 2022 [28]	A cross-sectional correctional descriptive study	Saudi Arabia	The aim was to describe the nurses’ knowledge, education, belief/attitudes, and implementation of SSC as well as to determine any relationships between them		Convenience sampling	Findings showed that nurses had a moderate level of knowledge, positive attitudes/beliefs, moderate education, and moderate implementation levels. The findings also revealed a significant association between nurse’s knowledge, attitude/beliefs, education about SSC, and nurses’ perceptions towards SSC implementation in a tertiary hospital	SSC knowledge, attitudes, and belief; SSC education; SSC implementation
Fatma et al. 2022 [29]	A quasi-experimental design	Egypt	The aim was to evaluate the effect of a kangaroo care educational program for mothers on weight gain of premature neonates in neonatal intensive care units		Purposive sampling of 50 mothers of preterm infants	88% of mothers in the study had an unsatisfactory level of knowledge in the pre-educational program, while 96% of them had a satisfactory level of knowledge in the post-educational program. 64% of the mothers had incompetent practices in the pre-educational implementation, while 58% of them had competent practices in the post-educational program implementation	Identify the importance of KMC Apply KMC steps Apply daily routine care (umbilical cord care)
Muttau et al. 2022 [30]	Prospective descriptive study, using qualitative and quantitative methods	Zambia	The aim of the study was to describe the implementation of a KMC model among preterm infants and its impact on neonatal outcomes at a tertiary hospital in Lusaka, Zambia		None	A total of 573 neonates were enrolled into the study. 13 extremely low-weight preterm infants who were admitted to the KMC room graduated to Group A (1000 g-1499 g with a median weight gain of 500 g. Of the 419 very-low-weight neonates, 290 remained in Group A, while 129 improved to Group B (1500 g-24999 g) with a median weight gain of 280 g. Of the remaining 89 low-weight neonates, one regressed to Group A, while 77 remained in Group B, and 11 improved to group C (> 2500 g) with a median weight gain of 100 g	Benefits of KMC; Appropriate KMC techniques; Position of newborn during breastfeeding; Importance of practicing good hygiene; Individual support
Habte et al. 2023 [31]	Cross-sectional study	Southern Ethopia	The aim of the study was to assess the compliance of postnatal mothers toward World Health Organization-recommended elements of KMC in Southern Ethopia		Mothers who gave birth to preterm or low-birth-weight infants were purposively sampled, using a single population proportion formula with a margin error of 5% and a 95% confidence interval	The mean practice score of KMC item was 5.12 with 2 and 10 as the minimum and maximum item scores. Place of residence, mode of delivery, birth preparedness and complication readiness plan, maternal knowledge of KMC, and place of delivery were identified as significant predictors of compliance towards key elements of KMC	KMC technique practice; Adequate exclusive breastfeeding; Placing the baby in SSC position; Maintaining good personal hygiene; Observing the baby; Gaining family support
Moran et al. 2023 [32]	A mixed-methods systematic review	High-income settings	The aim of this study was to explore the content, experiences and outcomes of an intervention designed to increase early SSC in high-income settings		A narrative synthesis used to synthesize both qualitative and quantitative findings was used	Database searches generated 1221 sources and two studies were identified via hand-search. Ten studies were included; (*n* = 7) were designed to improve SSC, following a caesarean section. The studies explored SSC prevalence and duration (*n* = 7), breastfeeding prevalence (*n* = 4), while six studies considered mothers and health professionals’ experiences of the intervention	PRECESS approach (Practice, Reflection, Education, and training, Combined with Ethnography for Sustainable Success) educational posters and weekly email reminders about the benefits and recommendations of SSC were distributed Orientation training was provided to mothers regarding SSC
Samsidin et al. 2023 [17]	A quasi-experimental and longitudinal study with pre and post-intervention design	Malaysia	The aim of the study was to investigate the effects of a locally contextualized, structured kangaroo care education program on weight gain, breastfeeding rate, and length of hospitalization for premature infants		Forty-eight mother-infant dyads were purposively enrolled in the control and experimental groups	The kangaroo care hours performed by mothers at baseline in the experimental and control group was 4.12 and 0.55 h per week. At three months post-discharge, the experimental group had significantly higher weight gain, higher breastfeeding rates, and shorter lengths of hospitalization than the control group	Theoretical and practical demonstration of SSC practice; Provision of educational materials, for example, pamphlets

### Step 5: Presentation of findings

After synthesizing the data, it is recommended that a summary of the evidence should be written. In order to give a summary and interpretation of outcomes and characteristics of the included documents, the review usually provides both text and tables [20, 21]. The ILR report includes an integration of concepts, thoughts, definitions, or other relevant information that were derived from the included documents on the phenomenon being studied. Concluding statements are derived from the analysis and discussion of the synthesized information [21, 33]. The authors further critically analyzed and produced three main themes on the essential components of an educational program for implementing SSC for preterm infants as follows:

#### Theme 1: Policy and role players regarding the implementation of SSC

The institution should formulate a policy regarding the implementation of SSC. The written policy documents should be accessible to the nursing staff involved in the program and should be easy to apply [27]. Posters summarizing the policy of the institute should be displayed in the centre so that people are aware of SSC, along with its benefits and implementation methods [27].

A study conducted in Indonesia [25] aimed to assess the skills of neonatal nurses one year after training found that the development of kangaroo care services in the hospital not only provided training for health personnel but also required continuation, a clear policy and a Standard Operating Procedure (SOP). Therefore, having a policy about the SSC program enhances teaching and reinforces knowledge regarding it [25].

#### Theme 2: Education and training on the implementation of SSC

Health education is very important to both nursing staff and parents of preterm infants, and more time should be devoted to SSC activities. It is fundamental for them to comprehend what SSC entails and what its benefits are. In a study conducted in Zambia, mothers accepted SSC after they were given information and education through proper communication [27]. Providing education is an effort to improve knowledge levels of healthcare professionals regarding evidence-based interventions: exclusive breastfeeding, effective parenting strategies, and SSC in preterm infants [25].

A study conducted by Samsudin et al. [17] supported the application of a structured teaching program. They found that a planned teaching program on SSC was a successful method for reducing stress, producing a positive perception and good knowledge of the implementation of SSC [17]. Therefore, continuing education for healthcare professionals and parents is necessary.

A study conducted in Mexico highlighted that nurses and parents of preterm infants need to be trained through lecture methods, which covered the benefits and methodology of SSC and early breastfeeding, and incorporated recommendations based on the baby-friendly hospital initiative [12]. In addition, it was found that multiples trainings should be carried out to ensure that any challenges that arise regarding the implementation of SSC can be dealt with [12]. In order to reduce neonatal morbidity and mortality due to premature birth, it is important need to increase the knowledge and skills not only for mothers but also healthcare professionals.

Furthermore, proper training for healthcare professionals and the creation of a welcoming environment for parents are also essential elements for effective implementation of SSC [32]. The program should focus on increasing awareness and knowledge about SSC, ensuring enough knowledge and practice about SSC and continuous educational programs to increase awareness about SSC [32]. Therefore, it is important to emphasize to the mothers of preterm infants that promoting and practising SSC is a cost-effective intervention with social and economic benefits [24].

#### Theme 3: Counselling and support for healthcare professional and parents of preterm imfants

Counseling and support for mothers and neonatal nurses as it relates to SSC for preterm infants is crucial for improving outcomes. Nurses play a significant role in providing support to mothers in intensive care units and should be equipped with strategies to reduce maternal stress [34]. Mothers of preterm infants perceive nursing support as encompassing the delivery of information, professional care and emotional support [34]. Furthermore, nurses should have a comprehensive understanding of parents' education and support needs, and standardized tools should be used to identify these needs [35]. A study conducted in Southern Ethopia concluded that mothers should be counseled during antenatal care and after delivery to improve their knowledge regarding SSC [31].

## Discussion

The goal of this integrative literature review was to identify, synthesize and present findings on essential components of an educational program to implement SSC for preterm infants in intensive care units. The study highlighted the essential components of an educational program to implement SSC for preterm infants. They include policy formulation and role players for the implementation of SSC, education and training as well as counseling and support nurses and mothers of preterm infants.

The evidence supports the importance of policy implementation and the involvement of various role players, including nursing staff and parents, in promoting and facilitating SSC for preterm infants. This finding concurs with the study conducted by Nuraini et al. [25] who concluded that the development of SSC leads to the clear formulation of policy and SOP.

The included studies revealed that structured education on the evidence-based practice and benefits of SSC helps to overcome a lack of knowledge regarding SSC for parents of preterm infants and healthcare professionals. An educational program was found to be effective in improving parental knowledge in caring for preterm infants in a district hospital in neonatal intensive care unit in Kigali [14, 29]. Therefore, the findings of a study can serve as an instructive demonstration for healthcare professionals and parents who are able to effectively employ SSC and enable the advantages to be obtained by preterm infants. In addition, a study conducted by Herzberg et al. [34] indicated that the nurse manager plays a role in providing support and opportunities for ongoing education.

The findings from this study confirmed that more research is needed on educational programs in order to reduce mortality and morbidity rates among preterm infants [36]. Despite recent changes in the provision of health care for preterm infants, nurses still experience several barriers in successfully implementing SSC in the healthcare settings. The review conducted by Maniago et al. [26] reported strategies to reduce barriers and to improve utilization of SSC as clear guidelines, sufficient supplies and equipment, capacity building among staff and proper information dissemination for parents regarding SSC. Moreover, after the implementation of advocacy, training, and promotion of intermittent SSC to premature infants, it was well-received by their parents [28, 37].

By identifying the essential components for implementation of SSC, it becomes possible to design an educational program that can support the continued growth and development of premature infants and the overall well-being of parents. This can be achieved more quickly by including the parents’ and nurses’ inputs to ensure their needs are incorporated into the program. Therefore, this study recommends the continuous promotion of SSC education as proven to be safe and efficacious in managing preterm infants. Additional research and investigations are warranted to ascertain the effectiveness of various educational methods with regard to enhancing knowledge and practice related to SSC for parents of preterm infants. The urgency for research in the domain of awareness and practice is thus underscored.

### Limitation

This study has several limitations. The current ILR shows the absence of published studies in LMICs in Africa. Further research beyond the boundaries of high-income countries is required to determine the essential components to be incorporated in an educational program to implement SSC for preterm infants in LMICs. Moreover, only studies published in English were selected for inclusion due to limited resources for translation services. Therefore, some studies published in other languages may provide varying or more diverse perspectives on the essential components of such an educational program. Despite these limitations, the current study's findings can serve as a foundation for the development of an educational program that targets healthcare professionals and parents of preterm infants in intensive care units.

## Conclusion

A comprehensive literature search spanning the recent literature identified twelve studies on essential components of an educational program for implementing SSC for preterm infants in intensive care units. The integrative review captured the following essential components: policy and role players on implementation of SSC, education and training as well as counseling and support needs for nurses and parents of preterm infants. The results of this study could aid program implementers, policy makers, and researchers to implement and scale up this important tool of SSC in intensive care units and its potential to improve breastfeeding practices. Further research regarding educational program for implementing SSC for preterm infants in intensive care units is warranted.

## Data Availability

The dataset used and analysed during the current reviews are available from the corresponding author on reasonable request.

## Abbreviations

CASP: Critical Appraisal Skills Program
ILR: Integrative Literature Review
KMC: Kangaroo Mother Care
LBW: Low Birth Weight
LMICs: Low-to-Middle Income Countries
PIOT: Population, Intervention, Outcome and Time frame
NWP: North West Province
SA: South Africa
SOP: Standard Operating Procedure
SSC: Skin to Skin Contact
WHO: World Health Organization
